# Investigation into the Toxicity of Traditional Uyghur Medicine Quercus Infectoria Galls Water Extract

**DOI:** 10.1371/journal.pone.0090756

**Published:** 2014-03-07

**Authors:** Mubarak Iminjan, Nurmuhammat Amat, Xiao-Hui Li, Halmurat Upur, Dilnur Ahmat, Bin He

**Affiliations:** 1 XiangYa Hospital, Central South University, Changsha, Hunan, People's Republic of China; 2 Pharmaceutical Science Institute, Xinjiang Medical University, Urumqi, Xinjiang, People's Republic of China; 3 Traditional Uyghur Medicine Institute, Xinjiang Medical University, Urumqi, Xinjiang, People's Republic of China; 4 Department of Pharmacy, Central South University, Changsha, Hunan, People's Republic of China; 5 Department of Pharmacy, Xinjiang Uyghur Autonomous Regions Petroleum Hospital, Urumqi, China; 6 Department of Pharmacy, Xinjiang Uyghur Autonomous Regions people's Hospital, Urumqi, China, Xinjiang, People's Republic of China; University of Kentucky, United States of America

## Abstract

**Objective:**

Quercus infectoria galls (QIG) is being widely used in Traditional Uyghur Medicine. To gather preclinical safety information for the aqueous extract of QIG, a toxicity study was performed.

**Methods:**

Subject animals were randomized, and devided into exposure and control groups. In the acute toxicity phase, three different doses—5, 7.5, and 10 g/kg, respectively—were administered via enema to imprinting control region (ICR) mice. An experiment using the maximum tolerance dose (MTD) i.e.10 g/kg was also performed. Data were gathered for 14 days, and study parameters were clinical signs, body weight, general behavior, adverse effects and mortality. At the day 14, major organs of the subjects were examined histologically. Chronic toxicity was also evaluated in Wistar rats for over 180 consecutive days. The rats were divided into three groups with different doses of 0.2 g/kg, 0.8 g/kg, and 2 g/kg, QIG. Furthermore, observations were carried out in rabbits to investigate if there were signs of irritation.

**Results:**

In comparison to control group, acute, chronic toxicity and mortality were not significantly increased in exposure group.

**Conclusion:**

Study result suggests that the aqueous extract of QIG is unlikely to have significant toxicity and that clinical trials may proceed safely.

## Introduction

The galls of the *Quercus infectoria Olivier* plant (also known as the Gall Oak or *Quercus Lusitanica* in the family Fagaceae) grow as a result of infections of trees or shrubs by the *Cynips gallae tinctoriae* wasp. These plants are mainly found in Greece, Asia Minor, Syria and Iran. *Quercuse infectoria* galls (QIG, also known as *Galla Turcica*) are known to have multiple therapeutic properties and used widely in several traditional medicine as an astringent or an anti–inflammatory agent [1], [2]. Pharmacologically, QIG has demonstrated various effects such as astringent, antiparkinsonian, anti-tumor, antidiabetic, local anesthetic, antipyretic, antioxidant, antimicrobial, antibacterial and antifungal activity [3]–[7]. QIG is consisted of a large amount of tannins (50%∼70%), gallic acid, syringic acid, ellagic acid, sitosterol, amentoflavone, hexamethyl ether, isocryptomerin, methyl betulate, methyloleanate and hexagalloyl glucose [8]–[10].The chemical components of QIG include five main substances such as gallic acid, m-digallic acid, methyl gallate,1,2,3,6-tetra-O- galloyl-β-D-glucose and 1,2,3,4,6-Penta-O-galloyl-β-D-glucose. Therefore, a quality control standard for QIG has been established [11].

In Central Asia, the versatility of QIG has made it one of the most popular plants in Traditional Uyghur Medicine (TUM) over a thousand years. QIG has been used as an astringent, a moisture eliminator, an anti-inflammatory agent (i.e., to treat erysipelas), an antiseptic and an antidiarrheal agent. In TUM, QIG is most commonly seen in the treatment of intestinal dysmotility, dysentery, functional enteritis, hemorrhagic sores, alopecia areata, dental caries, periodontitis, halitosis, pharyngolaryngitis and tympanitis [12]. One of the most common uses of QIG is in the treatment of Ulcerative Colitis (UC) as an enema. QIG has been reported that it has anti-inflammatory properties. For example, in UC models it showed the ability to down-regulate cyclooxygenase-2 (COX-2), IL-6, c-jun and iNOS expression [13]–[16]. It has also been reported that QIG decreases ADP-induced platelet aggregation in rats with UC[17].

However, there have been no reports published about acute or chronic toxicity of QIG. The purpose of this study was to evaluate the possible toxicity of QIG aqueous extract via biochemical, hematological and histopathological parameters. Then, study results can provide clinicians about safe levels of QIG doses. Acute effect and mucosal irritation were evaluated in mice and rabbits following the administration of QIG per rectum. In addition, chronic adverse effects of QIG were also studied in rats.

## Materials and Methods

### Preparation of the QIG aqueous extract

Air-dried samples of QIG were purchased from the Xinjiang Autonomous Region Traditional Uyghur Medicine Hospital (Urumqi, China) and their authenticity was verified by a qualified pharmacist. Three kilograms of the gall powder were soaked in distilled water, at a volume ratio of 1∶8, for 1 hour. After this, the aqueous extract was brought to a boil point over three consecutive 30-minute periods. The extract was then put through a plate-and-frame filter press before being transferred to a reduced pressure, low temperature (<60°C) rotary evaporator to achieve a concentration of 40 mg/L (W: V). The drug was examined and standardized according to general quality control standards of the current Chinese Pharmacopoeia [18] and according to our previously reported analytic methods [19].

### Preparation of Test Animals

We used Specific Pathogen Free (SPF) animals. ICR mice (20±2 g) were chosen for the acute toxicity study. Wistar rats (200±20 g) were used by our group in the past for UC studies [11], [13]–[17], [20]. New Zealand Rabbits (2.5±0.5 kg) were selected for evaluating the degree of mucosal irritation from the extract due to their sensitivity. The qualification certificate number of animals was SCXK (XIN) 2003-0001, and they were all procured in the Xinjiang Medical University laboratory animal center. The test subjects were kept in environmentally controlled cabinets. The air temperature was maintained at 23±2°C and humidity at 50–60%. They were kept under a 12-hour light–dark cycle (07:00–19:00) fed with food pellets and water ad libitum. The components of the mouse and rat feed were wheat, corn, bean pulp, fatty residue and fish flour. Rabbits were fed with grass powder. The protocols for these experiments were approved by the Xinjiang Medical University Ethics Committee on Animal Experimentation and conducted in accordance with internationally accepted principles for the use and the care of laboratory animals.

### Administration of QIG Aqueous Extract

#### Acute toxicity study

The ICR mice were randomly assigned and divided by gender to experimental groups of ten mice each and a control group of ten mice. The entire experimental group consisted of 15 male and 15 female mice. The control group consisted of 5 male and 5 female mice. These animals were given distilled water enemas. Three experimental groups of ten mice each were assigned to receive QIG aqueous extract in doses of 5, 7.5 and 10 g/kg body weight, respectively, via enema. These doses are respectively equivalent to 150, 225 and 300 times the usual therapeutic dose for an adult human (60 kg). At the highest dose, mice received 5 mL of fluid per rectum at a concentration of 40 grams of plant material per liter.

Immediately after administration, food and water were withheld from the mice and were observed for signs of toxicity at intervals of 15, 30, 60, 120 and 240 minutes. Beyond this, the mice were observed daily for behavioral changes and physical signs of toxicity or death for up to 7 days [21].

Because none of the mice died in this part of the study, the LD_50_ could not be determined. For this reason, a maximum tolerance dose (MTD) experiment was carried out.

For the MTD study, ICR mice were randomly assigned to a control group and an experimental group of 20 mice each. QIG aqueous extract was administered rectally to the experimental group in single doses of 10 g/kg of body weight. The mice were observed daily for behavioral changes and physical signs of toxicity or death for up to 14 days afterwards.

#### Mucosal irritation study

New Zealand male rabbits were randomly assigned to three groups: a control group receiving distilled water enemas (n = 4), the first exposed group receiving 0.1 g/kg of QIG aqueous extract enemas (QIG1, n = 9) at a concentration of 0.04 g/ml, and the second exposed group receiving 0.95 g/kg of QIG aqueous extract enemas (QIG2, n = 4) at a concentration of 0.38 g/mL. Animals in each group received enemas in volumes of 2.5 mL/kg for 14 consecutive days. During this period, food and water intake as well as daily body weight measurements were taken. Afterwards, the rabbits were sacrificed, and colon and anal tissue samples were collected for Hematoxylin-Eosin (HE) staining and histological examination. A standardized table was used to grade histological changes and scores were used for quantitative analysis (Grading of Pathologic Changes for the Rabbit Mucosal Irritation Experiment see Table 1).

**Table 1:** Standard for Grading pathologic change in mucosal tissue samples.

Morphological Changes	Severity	Grade
No change or no significant change	No irritation	0∼0.40
Mild hyperemia, mild secretions	Mild irritation	0.41∼1.5
Moderate congestion, increased secretions	Moderate irritation	1.51∼2.50
Moderate congestion, edema, copious secretions, mucosal deformation	Severe irritation	≥2.51

#### Chronic toxicity study

Wistar rats were randomly assigned to four groups of 30 animals each, equally divided by gender. This first group was a control group that received distilled water enemas. Groups 2 to 4 (QIG1 to QIG3) received daily QIG aqueous extract enemas in doses of 0.2, 0.8 and 2 g/kg (5 mL/kg volumes) for 180 days. After this period, the rats were observed for 30 more days of convalescence. Daily observations were made regarding signs of toxicity, mortality, water and food consumption and body weight. At intervals of 90, 180 and 210 days, ten rats in each group were set aside, made to fast for 12 hours and then anesthetized with pentobarbital. Under anesthesia, blood samples were collected from their abdominal aortas for hematology evaluation (in a tube containing EDTA anticoagulant), biochemical testing (no anticoagulant) and prothrombin time (tube with sodium citrate) testing. The blood used for biochemical testing was allowed to clot before centrifugation at 3,000 rpm and 4°C for 10 min. The serum was then collected and analyzed.

### Hematology and biochemistry

Hematological analysis was performed using a hematology analyzer (LH 750, Beckman Coulter). Indices that were recorded included: hematocrit (Hct), hemoglobin (Hb), red blood cell count (RBC), mean corpuscular volume (MCV), mean corpuscular hemoglobin (MCH), mean corpuscular hemoglobin concentration (MCHC), white blood cell count (WBC), red blood cell volume distribution width (RBC%), neutrophil count (N), lymphocyte count (L), monocyte count (M), eosinophil count (E), basophil count (B) and platelet count.

Serum biochemical analysis was performed with an automated chemistry analyzer (LX20, Beckman Coulter). Recorded measurements included: alkaline phosphatase (ALP), alanine aminotransferase (ALT), aspartate aminotransferase (AST), blood urea nitrogen (BUN), total protein (TP), albumin (ALB), bilirubin (BIL), creatinine (Cr), glucose (GLU), uric acid (UA), triglycerides (TG), total cholesterol (TC), sodium (Na), potassium (K) and chloride (Cl).

Blood coagulation measurements were performed with an automated coagulation analyzer (ACL Advance, Beckman Coulter). Thrombin time (TT), prothrombin time (PT), activated partial thromboplastin time (APTT) and fibrinogen (FBG) measurements were taken.

### Histopathology

The brain, heart, kidneys, lungs, stomach, liver, spleen and uterus of test animals were examined. Observations were made *in situ* of each organ's position, shape, size and color as well as for any gross lesions. Then the organs were carefully dissected out and weighed. Fresh tissue slices from each organ were obtained then fixed in a buffered formaldehyde solution (10%), fixed with ethanol–benzene and enclosed in paraffin. A microtome was used to cut micrometer sections that were then prepared as slides and stained with HE. These samples were tested under light microscope examinations by a pathologist in a blinded fashion. Histological changes were graded according to Table 2 (Grading Scale for Determining Pathological Severity of Features Found in Tissue Samples from the MTD and Chronic Exposure Experiments see Table 2) and scores were used for quantitative comparisons.

**Table 2:** Scale for tissue sample pathology grading.

	Surface Appearance					
**Degree express**	0	≤5%	5%≥	25%≥	50%≥	≥75%
Sign	−	±	+	++	+++	++++
Numeric	0	0.5	1	2	3	4
Description	None	Minimal	Mild	Altered	Moderate	Severe

### Statistical Analyses

Statistical calculations for the data were performed using SPSS 17.0 software package. Comparisons were made using analysis of variance (ANOVA). Significance declared at P<0.5 and measured with the Student's T-test. All data are expressed as mean ± standard error of measurement (SEM).

## Results and Discussion

### Acute toxicity study

In the animals that received QIG aqueous extract, there were no deaths or any significant changes in behavior detected (Table 3). As a result, LD_50_ can not be calculated.

**Table 3:** Signs of acute toxicity test.

Dose(g/kg Body Weight)	Mortality		
	**D/T**	**Latency (h)**	**Toxic Signs**
5.00	0/10	-	None
7.50	0/10	-	None
10.0	0/10	-	None

Subsequently, results of the MTD experiments showed no statistical difference in body weight between the control and experimental groups (P>0.05). In addition, no gross signs of toxicity in any of the mice organs were found at necropsy (data are not shown here, but available upon request). The dose used for this phase of the study was more than 300 times the usual therapeutic dose in humans. Therefore, the MTD for QIG aqueous extract was observed to be greater than 10 g/kg.

**Figure 1:**
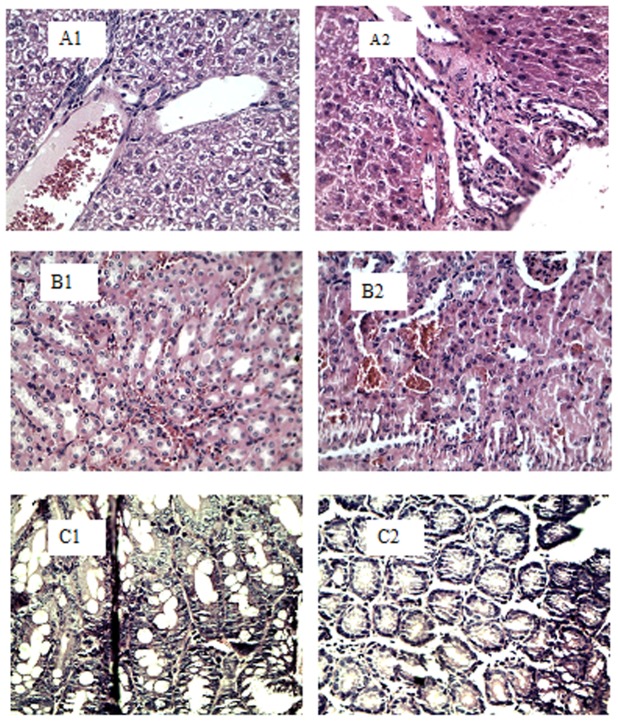
Histological samples from the MTD test: A–C. Panels A1–A2 show normal liver. Panels B1–B2 show normal kidney and Panels C1–C2 show normal colon tissues. **Control (×40), QIG3 (×40)**.

Microscopic examination revealed significant differences in Kupffer cell hyperplasia in the liver tissues of the mice (P<0.05). The experimental group had lower levels of hyperplasia than in the control group, as shown in Figure 1, A1–A2. Exposed mice had higher levels of minor edema than control mice in renal tubular epithelium (P<0.05, Figure 1, B1–B2). Intestinal mucosal epithelial hyperplasia was seen in both exposed and control groups, but less hyperplasia was seen in exposed mice than control mice, (P<0.05, Figure 1, C1–C2). No other statistical differences were noted for any other histological findings.

### Mucosal Irritation study

**Figure 2:**
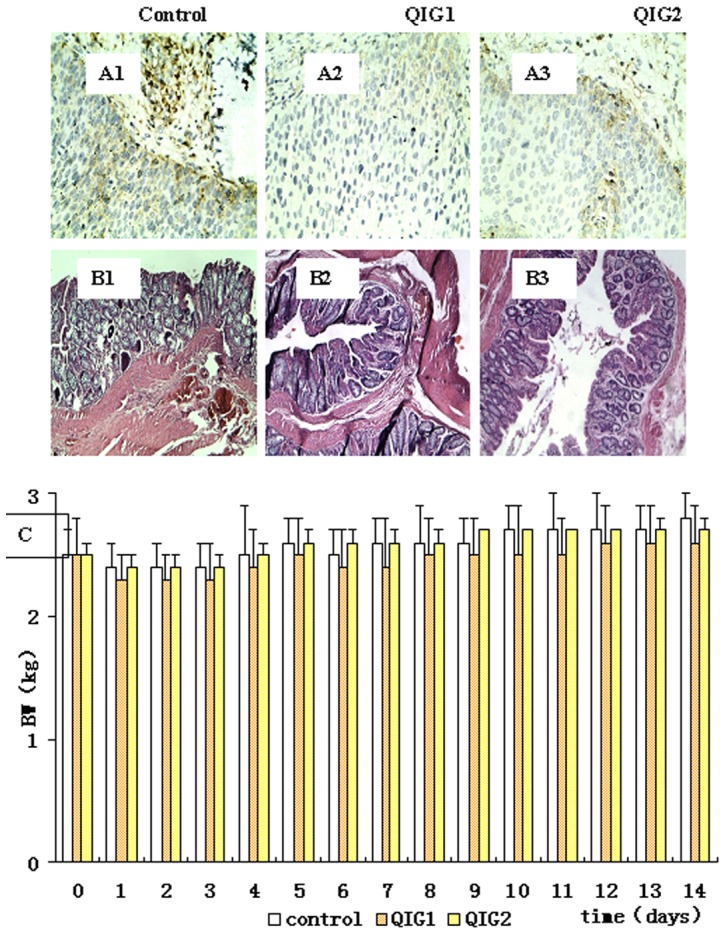
Histological and body weight changes in mucosal irritation test: A–C. Panels A1–A3 show photomicrographs of the colon sections (×10).Panels B1–B3 show photomicrographs of the anus sections (×40). Panel C shows body weight changes.

Daily rectal administration of QIG aqueous extract for 14 days did not produce any obvious sign of clinical toxicity in rabbits. After the administration of the enema at the beginning of the study, all the subjects reacted with signs of discomfort-such as attempting to withdraw, and anal spasms. Their behaviors were returned to normal several days later. No congestion or edema was observed in the colonic mucosa and anal tissues of the animals. As shown in Figure 2, C, there was no statistical difference in body weight between the experimental and control group; Intestinal epithelial hyperplasia was observed in all three groups, but there were no significant differences between them. There was a slight preponderance of gland cells in the serosal and muscular layers of the anal tissues of the control group, but this was not statistically significant (Figure 2, A–B).

### Chronic toxicity study

#### Effect of QIG extract on the body weight and feed consumption of rats

**Figure 3:**
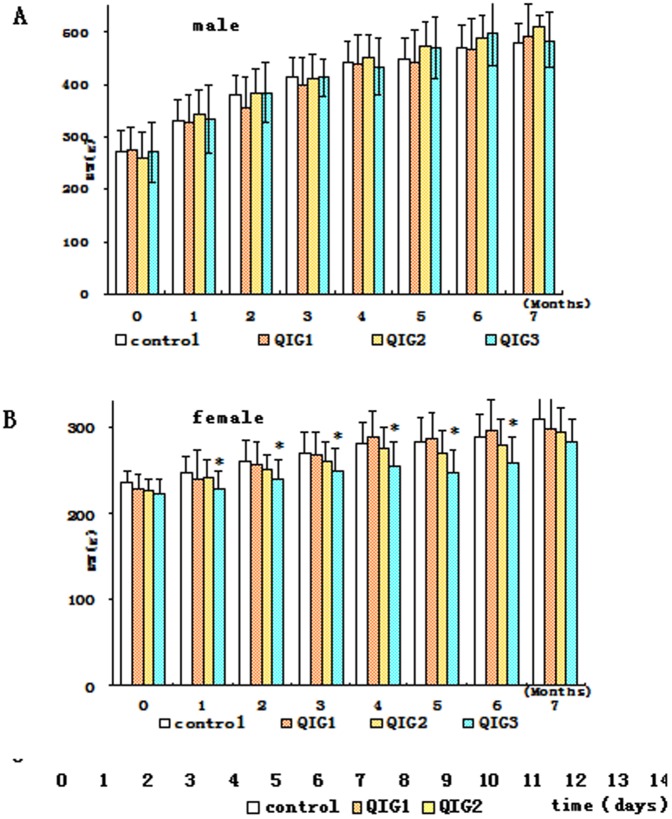
Body weight changes during chronic toxicity test: A–B. Panel A for male rats. Panel B for female rats.

Rectal administration of QIG aqueous extract for 180 consecutive days did not produce any obvious signs of toxicity or any animal death, even at the highest dose of 2 g/kg. Changes in body weight are shown in Figure 3. There were no significant differences in changes in body weight between exposed and control groups. There were some trends seen in weight changes in different gender. However,at the lowest dose (group QIG1), differential weight gain was 0.6% for males and 2.5% for females compared to controls. At the other doses, these differences were 2.1% for males and 0.9% for females (QIG2) and 5.6% for males and 1.2% for females (QIG3). Overall, male rates showed greater weight gain than females. These changes varied by weight. The male rats gained more weight with higher doses, but females showed an inverse relationship between dose and weight change. Over six months, the difference in weight change between female rats receiving the highest dose (QIG3) and that of controls was statistically significant (P<0.05). These trends in weight changes are significant in female rats between QIG1 and QIG3 in 1, 2, 4, 5, 6 months; between QIG2 and QIG3 there were no difference. From the second week of 6 month these differences were not significant.

Overall food consumption was similar between the groups. Food intake generally decreased over the course of QIG administration, but there were no statistically significant changes throughout and into the convalescent stage, as shown in Table 4.

**Table 4:** Feed consumption of rats during chronic toxicity test*.

	Males (n = 15)Females (n = 15)							
Month	Dose (g/kg Body Weight)							
	**0**	**0.2**	**0.8**	**2**	**0**	**0.2**	**0.8**	**2**
**0**	51.00	47.24	51.41	48.92	49.77	50.79	46.15	48.95
**1**	38.38	42.01	40.05	39.47	36.83	36.17	37.09	39.64
**2**	38.54	41.88	39.32	41.10	40.56	41.47	41.07	39.63
**3**	35.62	40.23	37.21	34.77	37.69	38.96	39.86	41.84
**4**	34.53	36.42	38.20	40.62	40.02	39.05	39.64	44.57
**5**	37.51	42.38	47.19	38.87	47.65	42.09	45.58	44.94
**6**	38.74	41.20	42.20	38.65	48.15	46.48	48.15	56.01
**7**	42.41	41.79	43.11	41.72	47.90	49.80	51.42	55.18

*Values represent the mean for each group.

#### Effect of QIG extract on the hematological parameters and biochemical parameters of rats

Hematologic and biochemical data showed significant differences between groups in MCV, MCHC, WBC, RDW and ALT., male rats received the medium dose had lower MCV and higher MCHC readings than the control group (P<0.05). However, these changes were not dose-dependent, and no similar level of statistical difference was seen in female rats. Female rats had a higher WBC in the medium dose group and a higher RDW in the lower dose groups than controls, but this was not seen in males, as shown in Table 5. Similarly, lymphocyte counts for females, but not male, were significantly elevated for exposed animals.

**Table 5:** Hematologic measures during the chronic toxicity test.

	Males (n = 15				Females (n = 15)			
**Parameter**	**Dose(g/kg)**							
	**0**	**0.2**	**0.8**	**2**	**0**	**0.2**	**0.8**	**2**
Hemoglobin(g/L)	141.8±5.4	135.6±2.1	141.7±7.0	137.1±11.2	135.8±6.4	142.3±9.4	142.0±6.3	147.7±19.7
RBC(×10^12^/L)	7.6±0.2	7.4±0.3	7.7±0.4	7.5±0.7	7.0±0.5	7.2±0.4	7.3±0.3	7.6±1.0
NRBC(%)	2.8±4.3	0.9±1.2	9.4±22.1	1.2±1.1	0.0±0.0	8.6±12.4	0.0±0.0	0.0±0.0
NRBC (×10^9^/L)	1.5±3.4	0.0±0.0	0.2±0.4	0.4±1.1	0.4±0.6	0.1±0.2	0.0±0.0	0.0±0.0
MCV (fL)	56.3±1.5	54.4±1.9	54.3±1.0	55.2±1.6	59.0±2.2	57.9±1.4	57.8±0.8	58.6±3.1
MCH (pg)	18.8±0.4	18.3±0.6	18.5±0.2	18.3±0.5	19.4±0.7	19.7±0.5	19.6±0.2	19.5±0.4
MCHC(g/L)	333.6±4.3	336.8±3.1	340.5±2.5*	331.0±4.5	328.3±9.2	341.0±3.0*	338.7±2.5	333.7±13.1
RDW (%)	15.1±0.9	15.7±1.2	16.0±0.6	15.7±1.1	12.7±0.8	14.0±1.0*	13.5±0.4	13.3±0.2
Platelet (×10^9^/L)	828±93	874±153	887±63	836±168	959±114	897±201	1033±116	1048±21
PT(sec)	20.9±4.1	23.9±2.2	24.1±1.8	23.8±0.7	22.5±1.6	21.9±1.1	23.2±1.5	22.6±0.0
APTT(sec)	22.8±2.1	25.3±1.1	21.5±1.8	20.1±2.7	20.2±3.3	20.1±2.7	21.2±1.9	20.0±1.7
FBG(g/L)	2.0±0.5	2.0±0.4	2.3±0.4	2.4±0.3	1.3±0.3	1.5±0.4	1.3±0.2	1.0±0.1
WBC(×10^9^/L)	2.1±1.0	1.4±0.7	1.6±1.0	1.3±0.7	1.5±1.6	2.6±1.4	4.7±3.0*	4.6±3.0
Neutrophils(×10^9^/L)	0.7±0.6	0.3±0.3	0.2±0.1	0.1±0.2	0.5±0.5	0.7±0.7	1.4±1.9	1.3±1.1
Lymphocytes(×10^9^/L)	1.4±0.7	1.0±0.8	1.3±1.0	0.9±0.5	1.0±1.0	1.8±0.9	3.2±1.6*	3.3±1.9*
Monocytes (×10^9^/L)	0.02±0.04	0.00±0.00	0.00±0.00	0.00±0.00	0.00±0.00	0.00±0.00	0.00±0.00	0.00±0.00
Eosinophils(10^9^/L)	0.05±0.03*	0.01±0.01	0.02±0.01	0.02±0.01	0.02±0.02	0.04±0.04	0.07±0.07	0.06±0.09
Basophils (×10^9^/L)	0.00±0.01	0.07±0.15	0.10±0.24	0.23±0.28	0.00±0.01	0.01±0.01	0.05±0.12	0.02±0.02

Values are mean ± SD. *P<0.05 for difference from controls.

Female rats had significantly higher ALT levels than controls in the higher dosage group (P<0.05), as shown in Table 6. In addition, higher levels of ALP were seen in female rats received the medium and high doses. All of the blood values measured during this experiment was within their normal ranges.

**Table 6:** Biochemistry parameters in chronic toxicity test.

	Males (n = 15)				Females (n = 15)			
**Parameter**	**Dose(g/kg)**							
	**0**	**0.2**	**0.8**	**2**	**0**	**0.2**	**0.8**	**2**
AST (U/L)	164.5±64.7	126.9±41.4	125.1±30.4	118.0±23.8	75.9±35.6	101.5±18.3	102.3±25.7	105.5±9.3
ALT (U/L)	57.1±22.5	38.5±9.3	43.5±11.5	39.1±5.9	29.9±7.5	26.3±11.0	31.7±4.4	41.7±6.5*
ALP (U/L)	96.3±10.8	90.7±8.8	91.1±22.4	96.2±23.4	45.8±6.1	41.1±8.0	72.4±24.6*	82.7±34.8*
Sodium(mmol/L)	140.6±0.9	145.6±5.4	140.7±1.3	140.6±1.0	140.3±1.0	140.3±0.5	142.6±2.5*	142.0±1.1
Potassium(mmol/L)	4.8±0.4	4.6±0.3	4.7±0.2	4.5±0.2	4.2±0.3	4.6±0.3	4.1±0.5	5.3±1.5*
Chloride(mmol/L)	101.5±1.7	104.6±1.9*	103.4±1.0	103.9±2.6	104.9±1.6	104.7±1.1	106.9±1.4*	107.9±3.1*
Urea(mmol/L)	7.5±1.3	6.4±0.7	6.6±0.4	7.3±1.1	8.0±1.6	6.8±0.9	7.4±1.5	8.2±1.2
Bilirubin(µmol/L)	6.5±2.9	7.1±1.8	7.2±2.5	7.6±2.1	6.8±2.6	7.6±1.7	6.1±1.5	7.9±1.5
Creatinine(µmol/L)	51.4±6.5	44.9±3.4	49.7±6.8	47.1±4.4	42.2±6.2	42.7±4.4	44.6±3.6	43.3±3.1
Protein(g/L)	51.7±1.1	49.1±1.9	49.9±3.3	52.6±7.9	63.1±11.3	55.1±1.3	52.5±3.5	55.3±3.1
Albumin(g/L)	14.8±1.0	14.4±0.7	14.2±0.6	15.1±0.9	19.1±2.0	19.2±1.2	17.6±1.8	19.7±1.9
Globulin(g/L)	36.9±0.6	34.7±1.5	35.8±3.1	37.5±7.1	44.1±11.6	36.0±2.2	34.9±2.0	435.7±1.4
AG ratio	0.40±0.03	0.42±0.02	0.40±0.03	0.41±0.05	0.46±0.14	0.54±0.06	0.50±0.04	0.55±0.04
Cholesterol(mmol/L)	0.9±0.2	0.9±0.2	0.9±0.0	1.1±0.5	1.9±1.2	1.2±0.1	1.1±0.2	1.2±0.1
Glucose(mmol/L)	8.7±2.8	7.1±0.6	8.3±1.4	8.2±0.4	7.5±2.3	6.1±0.7	6.4±0.6	5.3±1.2

Values are mean ± SD. *P<0.05 for difference from controls.

#### Histopathological changes

**Figure 4:**
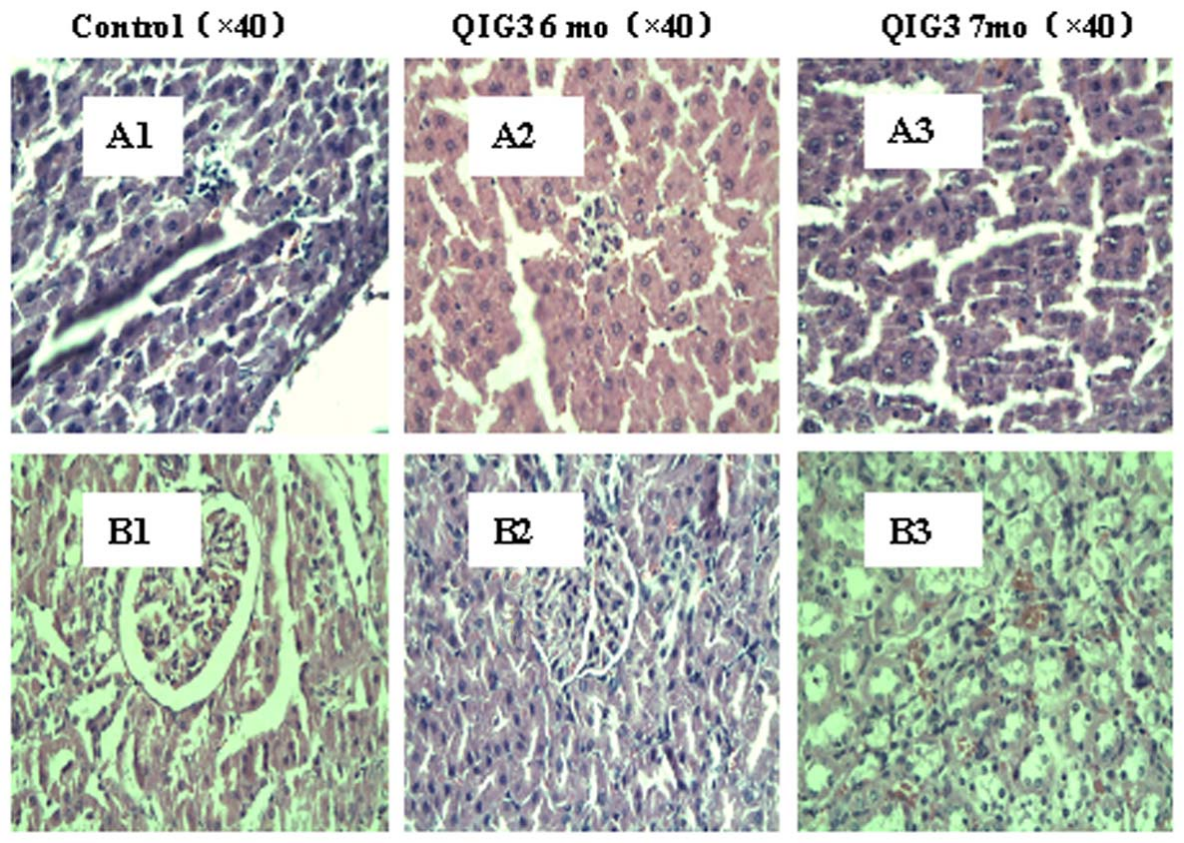
Histological examination during chronic toxicity test: A–B. Panels A1–A3 for liver and panels B1–B3 for kidneys tissues. **Control (×40), QIG3 6 mo (×40), QIG3 7mo (×40)**.

Histological changes in the liver at 6 and 7 months for control and exposed rats receiving QIG extract are shown in Figure 4. Both control and experimental animals showed signs of spotty necrosis and steatosis, but there was no statistical difference between them (Figure 4, A1–A3). Minor edemas in the renal tubular epithelium as well as slight glomerular capillary stasis were seen in all groups with no statistical difference as displayed in Figure 4, B1–B3. Histological changes in other organs also showed no statistical difference between controls and exposed rats as well as between different dose groups.

## Discussion and Conclusions

Plant-derived medicines continue to be used throughout the world, and many major drugs have historically been extracted from plants. Herbal medicines are commonly used in alternative medical practice [22]–[24]. The therapeutic use of plant products is increasingly popular as more consumers have faith in their benefits and in their purported absence of adverse effects [25]. However, the rationale for the utilization of medical plants has rested largely on experiences of clinical practitioners with little or no scientific data on their efficacy and safety [26].

To determine the safety of drugs for human use, toxicological evaluation is always firstly carried out with experimental animals to assess potential toxicity and to provide guidance on safe doses for human being [27]. The evaluation of chronic adverse effects may be more important in the determination of the overall toxicity of these drugs [28].

In this study of QIG aqueous extract, there were no mice died during the acute toxicity experiment despite receiving the highest dose (10 g/kg) that is 300 times greater than that usually administered in Traditional Uyghur Medicine. In addition, there were no significant adverse effects noted in the animal behavior. We did observe potential signs of an anti-inflammatory effect in those exposed animals compared to controls which was demonstrated via reduced levels of hepatic Kupffer cell proliferation and intestinal mucosal hyperplasia. We therefore, conclude that QIG unlikely has toxic effects in acute administration despite using extremely high doses.

To evaluate the local tissue effects of the QIG preparation, colon and anal tissues obtained from sensitive animals (rabbits) were examined. The apparent discomfort in rabbits with rectal manipulation was likely normal and resolved after the first few days. We found that there were no signs of congestion or edema in the colonic or anal mucosae of these animals, suggesting that such administration of QIG aqueous extract dose does not irritate these tissues. This conclusion was also supported by the histological findings. Although intestinal epithelial hyperplasia and anal glandular hyperplasia were detected, there were no significant differences between the groups. Our data show that QIG aqueous extract enemas did not cause significant local mucosal irritation.

Similar to acute administration, chronic use of QIG aqueous extract did not result in obvious toxicity. Rats were given QIG aqueous extract with doses from 0.2 g/Kg to 2 g/kg for up to 180 days. Although we noticed some changes in body weight and nutrient consumption as well as minor histological changes over the course of the study, none of these were significantly different between exposed and control animals. Furthermore, the observed weight of organs such as the brain, heart, kidneys, lungs, stomach, liver, spleen, and uterus were not significantly changed by exposure to QIG. Although our results suggest a high level of safety for the chronic use of QIG including a lack of appetite suppression, some of these preliminary histological findings may warrant further research to evaluate the adverse effects of chronic QIG aqueous extract administration.

Blood parameters are also important for evaluating toxicity since they have higher predictive values [29]. Chronic exposure of the rats to the highest dose (2 g/kg) of the QIG aqueous extract produced small and transient changes in some biochemical and hematological parameters such as MCHC. These changes were resolved by the end of the period of chronic exposure. However, we did see elevations in ALT and ALP in female rats but not in male rats. This could suggest a gender difference in response to QIG and further research should be carried out to verify these changes.

The rectal administration of QIG aqueous extract appeared to have very low, if any, toxicity. However, studies on experimental animals cannot always be relied upon to predict safety for human trials, additional studies are needed to define a safe and effective dose that is free of all toxic effects. In addition, it would be useful to investigate QIG toxicity in pregnant animals. The use of other animal models to evaluate toxicity, such as rabbits and guinea pigs may provide greater reassurance about the safety of this product in humans [30].

## References

[1] Chopra RN, Nayar SI, Chopra IC (1956) Glossary of Indian medicinal plant. C. S.I. R. New Delhi India.208: 255–256.

[2] Kaur G, Athar M, Alam MS (2008) Quercus infectoria Galls possess antioxidant activity and abrogates oxidative stress-induced functional alterations in murine macrophages. Chem Biol Interac171: 272–282. 10.1016/j.cbi.2007.10.002 18076871

[3] Everest A, Ozturk E (2005) Focusingon ethnobotanical uses of plants in Mersin and Adana provinces (Turkey) Journal Ethnobiology. Ethno medicine 1: 1–6. 10.1186/1746-4269-1-6 PMC1277086 16270936

[4] Hamid H, Kaur G, Abdullah ST, Ali M, Athar M, et al (2005) Two new compounds from the galls of Quercus infectoria with nitric oxide and superoxide inhibiting ability. Pharma Biol 43: 317–323. 10.1080/13880200590951711 28925837

[5] Yamunarani K, Jaganathan R, Bhaskaran P, Govindaraju R, Velazhahan R (2005) In vitro Antifungal Activity of a 29-kDa Glycoprotein Purified from the Galls of Quercus infetoria. Acta Phytopath Entomol Hungarica 40: 43–54.

[6] Hwang JK, Kong TW, Baek NI, Pyun YR (2000) Alpha-glycosidase inhibitory activity of hexagalloylglucose from the galls of Quercus infectoria. Planta Med 66: 273–274. 10821056 10.1055/s-2000-8569

[7] Dar MS, Ikram M (1979) Studies on Quercus infectoria; isolation of syringic acid and determination of its central depressive activity. Planta Med 35: 156–161. 419182 10.1055/s-0028-1097197

[8] Kaur G, Hamid H, Ali A, Alam MS, Athar M (2004) Antiinflammatory evaluation of alcoholic extract of galls of Quercus infectoria. J Ethnopharmacol 90: 285–292. 15013194 10.1016/j.jep.2003.10.009

[9] Dar MS, Ikram M, Fakouhi T (1976) Pharmacology of Quercus infectoria, J Pharm Sci. 65: 1791–94. 10.1002/jps.2600651224 1032663

[10] Ikram M, Nowshad F (1977) Constituents of Quercus infectoria. Planta Med31: 286–287. 10.1055/s-0028-1097531 866492

[11] Ren Yuan (2005) Study on chemical constituents of Turkish galls and Preparation Kuijie'an enema[D].Urumqi: Xinjiang Medical University.

[12] Wang JY, Zhang QH, Deng XM, Wang DC, Han WY (2007) Progress on pharmacological activities of Quercus infectoria galls. Shizhen Guoyi Guoyao 18: 2570–2572.

[13] Guo XY, Kurexi Y, Huang JJ, Huang GH, Yang M, et al (2009) Effect of Xi pa yi Kuijie'an on COX-2 gene expression in rat ulcerative colitis. Disi Junyida Xuebao 30: 501–504.

[14] Nassirhadjy M, KoreshY, Parhat A, DilbarW, Halmurat U (2007) The effection of QIG to rat IL-6 gene at mRNA level. Xinjiang Yike da Xuebao 29: 935–938.

[15] He J, Wufuer H, Huang JJ, Wu J, Yunusi K (2009) Xipayi Kui Jie'an downregulates c-jun expression in rat ulcerative colitis. World J Gastroenterol 15: 3441–3445.

[16] Huang JJ, Yunusi K, He J, Ni Q, Chen Y, et al (2010) Xipayi Kui Jie'an downregulates iNOS expression in rat ulcerative colitis. World J Gastroenterol 16: 350–354.

[17] Wu HL, Mao XM, Zhang FY, Halmurat U (2005) Effects of KuiJie'an on platelet agglomeration in rabbits and ulcerative colitis rats. Xinjiang Yikeda Xuebao 28: 35–37.

[18] National Pharmacopoeia Committee (2005) Pharmacopoeia of Peoples Republic of China [M]. Part 1 Beijing: Chemical Industry Press: Appendix VlB,VllG,XB,VlA,VlllC.

[19] Mubarak I, Dilnur A, Zeng CL, Halmurat H (2012) Study on the quality standard of Xipayi Kuijie'an Enema. China journal of hospital pharmacy 32: 1397–1399.

[20] Mubarak I, Kurax Y, Mawlanjan H, Halmurat H, Li YJ (2011) Experimental Study on Effects of Uyghur Mmedicine Xipayi Kuijie'an on Colon Mucosa Apoptosis and Mechanism of Treating Ulcerative Colitis. Science and Technology Review 29: 29–35.

[21] Twaij HAA, Kery A, Al Khazraji NK (1983) Some pharmacological, toxicological and phytochemical investigations on Centaurea phyllocephala. J Ethnopharmacol 9: 299–314. 6677820 10.1016/0378-8741(83)90037-5

[22] Cooper EL, CAM eCAM (2005) Bioprospecting: The 21st century pyramid. Evid Based Complement and Alternat Med 2: 125–127. 15937551 10.1093/ecam/neh094 PMC1142206

[23] Cooper EL (2004) Drug discovery, CAM and natural products. Evid Based Complement and Alternat Med 1: 215–217. 15841253 10.1093/ecam/neh032 PMC538505

[24] Tsao JCI, Zeltzer LK (2005) Complementary and alternative medicine approaches for pediatric pain: a review of the state-of-the-science. Evid Based Complement and Alternat Med 2: 149–159. 15937555 10.1093/ecam/neh092 PMC1142204

[25] Kouitcheu Mabeku LB, Penlap Beng V, Kouam J, Essame O, Etoa FX (2007) Toxicological evaluation of ethyl acetate extract of Cylicodiscus gabunensis stem bark (Mimosaceae). J Ethnopharmacol 111: 598–606. 17350192 10.1016/j.jep.2007.01.010

[26] Zhu M, Lew KT, Leung P (2002) Protective effects of plants formula on ethanol- induced gastric lesions in rats. Phytother Res 16: 276–280. 12164277 10.1002/ptr.839

[27] Brock WJ, Trochimowicz HJ, Millischer RJ, Farr C, Kawano T, et al (1995) Acute and sub-chronic toxicity of 1,1-dichloro-1-fluoroethane (HCFC-141b). Food Chem. Toxicology 33: 483–490. 10.1016/0278-6915(95)00008-p 7797175

[28] Qin Y, Wu X, Huang W, Gong G, Li D, et al (2009) Acute toxicity and sub-chronic toxicity of steroidal saponins from Dioscorea zingiberensis C.H.Wright in rodents. J Ethnopharmacol 126: 543–550. 19735710 10.1016/j.jep.2009.08.047

[29] Olson H, Betton G, Robinson D, Thomas K, Monro A, et al (2000) Concordance of toxicity of pharmaceuticals in humans and in animals. Regular Toxicol and Pharmacol 32: 56–67. 10.1006/rtph.2000.1399 11029269

[30] Onderdonk AB (1985) Experimental Models for Ulcerative Colitis. Dig Dis Sci12 Supp: 40S–44S. 10.1007/BF01296973 4064873

